# Abnormal Functional Connectivity of Anterior Cingulate Cortex in Patients With Primary Insomnia: A Resting-State Functional Magnetic Resonance Imaging Study

**DOI:** 10.3389/fnagi.2018.00167

**Published:** 2018-06-05

**Authors:** Chao-Qun Yan, Cun-Zhi Liu, Xu Wang, Jian-Wei Huo, Ping Zhou, Shuai Zhang, Qing-Nan Fu, Jie Zhang, Zhong-Yan Wang, Qing-Quan Liu

**Affiliations:** ^1^Department of Acupuncture and Moxibustion, Beijing Hospital of Traditional Chinese Medicine Affiliated to Capital Medical University, Beijing, China; ^2^Department of Acupuncture, Dongfang Hospital, Beijing University of Chinese Medicine, Beijing, China; ^3^School of Life Sciences, Beijing University of Chinese Medicine, Beijing, China; ^4^Department of Radiology, Beijing Hospital of Traditional Chinese Medicine Affiliated to Capital Medical University, Beijing, China; ^5^Beijing Key Laboratory of Basic Study on Traditional Chinese Medicine Infectious Diseases, Beijing Hospital of Traditional Chinese Medicine Affiliated to Capital Medical University, Beijing, China

**Keywords:** primary insomnia, voxel-mirrored homotopic connectivity, functional connectivity, anterior cingulate cortex, resting-state fMRI

## Abstract

**Background:** Recently, there have been many reports about abnormalities regarding structural and functional brain connectivity of the patients with primary insomnia. However, the alterations in functional interaction between the left and right cerebral hemispheres have not been well understood. The resting-state fMRI approach, which reveals spontaneous neural fluctuations in blood-oxygen-level-dependent signals, offers a method to quantify functional interactions between the hemispheres directly.

**Methods:** We compared interhemispheric functional connectivity (FC) between 26 patients with primary insomnia (48.85 ± 12.02 years) and 28 healthy controls (49.07 ± 11.81 years) using a voxel-mirrored homotopic connectivity (VMHC) method. The patients with primary insomnia and healthy controls were matched for age, gender, and education. Brain regions, which had significant differences in VMHC maps between the primary insomnia and healthy control groups, were defined as seed region of interests. A seed-based approach was further used to reveal significant differences of FC between the seeds and the whole contralateral hemisphere.

**Results:** The patients with primary insomnia showed higher VMHC than healthy controls in the anterior cingulate cortex (ACC) bilaterally. The seed-based analyses demonstrated increased FC between the left ACC and right thalamus (and the right ACC and left orbitofrontal cortex) in patients with primary insomnia, revealing abnormal connectivity between the two cerebral hemispheres. The VMHC values in the ACC were positively correlated with the time to fall asleep and Self-Rating Depression Scale scores (SDS).

**Conclusions:** The results demonstrate that there is abnormal interhemispheric resting-state FC in the brain regions of patients with primary insomnia, especially in the ACC. Our finding demonstrates valid evidence that the ACC is an area of interest in the neurobiology of primary insomnia.

## Introduction

Insomnia is a remarkably prevalent disorder, which is characterized by difficulty in initiating sleep, maintaining sleep and impaired daytime functioning (Edinger et al., 2004). Approximately 8–40% of the adult population reports insomnia symptoms at some point in their lives, and 8–10% of people suffer from chronic insomnia (Fernandez-Mendoza et al., 2012). Insomnia increases the vulnerability for psychiatric disorders, especially for anxiety and depression (Sivertsen et al., 2009; Baglioni et al., 2011). Complaints of sleep disturbances are very frequent in neurological disorders such as Parkinson's disease, Alzheimer's disease, stroke, epilepsy, traumatic brain injury, and multiple sclerosis (Mayer et al., 2011). Insomnia also causes a reduction in the quality of life, work productivity, and impairment in social function (Rosekind and Gregory, 2010). It has been recognized as a major public health issue, associated with increased societal burden and health expense (Staner, 2010). Despite the huge socio-economic impact and physical consequences of chronic insomnia, its neural substrate still remain poorly understood.

Prior studies have reported that the imbalance between left and right hemispheric activity and poor coordination may contribute to the pathophysiology of insomnia. Supporting this, electroencephalogram (EEG) study had demonstrated asymmetric interhemispheric EEG coherence in patients with insomnia (Kovrov et al., 2006). Structurally, a diffusion tensor imaging study demonstrated that patients with primary insomnia (PI) showed reduced fractional anisotropy in the body of the corpus callosum (CC) (Li S. et al., 2016). It is well known that the CC is the main collection of white matter bundles connecting both hemispheres so that both sides of the body can be coordinated, which plays a critical role in communication and coordination between the two cerebral hemispheres (Wang et al., 2013). Structural damage in the CC may affect the functional coordination between the cerebral hemispheres. Significantly increased region-to-region functional connectivity (FC) between the left amygdala and right inferior frontal gyrus was found in PI patients (Huang et al., 2012). Recent study have found interhemispheric FC alternations in individuals in the healthy participants with insomnia symptoms (Li X. et al., 2017). Thus, it is reasonable to expect that deficits of hemispheric interactions could shed light on the etiology and pathogenesis of insomnia. It would be meaningful to examine interhemispheric coordination in insomnia.

Resting-state functional Magnetic Resonance Imaging (rs-fMRI), has been applied to explore the neurophysiological mechanisms associated with insomnia owing to its noninvasive and task-free nature (O'byrne et al., 2014). Rs-fMRI uses the blood-oxygen-level-dependent (BOLD) signal as a neurophysiological index, which is sensitive to spontaneous and intrinsic neural activity within the brain (Zuo et al., 2010a; Baria et al., 2011). Thus, it can be used as an efficient way to directly the insomnia-related inter-hemispheric coordination alterations (Wang et al., 2013). Voxel-mirrored homotopic connectivity (VMHC) is an approach that measures the resting-state FC between each voxel in one hemisphere and its mirrored counterpart in the other hemisphere (Zuo et al., 2010b). FMRI-based VMHC provides a feasible way to observe the whole-brain homotopic connectivity alterations (Xu et al., 2014). Different strengths of VMHC of different symmetric regions could represent different characteristics of the information of interhemispheric for bilateral sensory integration and motor coordination (Stark et al., 2008).

In this study, we collected rs-fMRI data from PI patients and healthy controls. A systematic VMHC analysis was used to compare interhemispheric resting-state FC. We hypothesized that PI patients would show abnormal VMHC values compared with healthy controls, suggesting that the impairment of interhemispheric coordination may be related to the pathogenesis of PI. VMHC is only evaluates the voxel-mirrored regions of the two hemispheres. In order to investigate the impaired functional coordination between two hemispheres expect the symmetric regions, the seed-based resting-state analysis was further performed to map FC patterns between the seed region and whole contralateral hemisphere. We also evaluated whether clinical characteristics of PI were correlated with altered FC in PI patients.

## Materials and methods

### Ethics statement

The study was approved by the Research Ethical Committee of Beijing Hospital of Traditional Chinese Medicine Affiliated to Capital Medical University (reference: 2014BL-003-01).

### Participants

From September 2014 to September 2016, PI patients were recruited from outpatient clinics in the Department of Psychosomatic Medicine of Beijing Hospital of Traditional Chinese Medicine Affiliated to Capital Medical University, by displaying recruitment posters outside the clinics. All participants gave written informed consent prior to inclusion.

The inclusion criteria for PI patients were as follows: (i) the patients satisfied the definition of primary insomnia by the Diagnostic and Statistical Manual of Mental Disorders, 4th Edition; (ii) complaints of difficulty in falling asleep, maintaining sleep, or early awakening at the same time were present for at least 1 month; (iii) right-hand dominance; (iv) age from 25 to 60 years; (v) psychoactive medication were not taken for at least 2 weeks before and during the study. Criteria for exclusion were as follows: (i) other sleep disorders (e.g., hypersomnia or parasomnia), (ii) insomnia associated with specific reasons such as drugs, alcohol, or significant psychiatric history (e.g., bipolar disorder, schizophrenia); (iii) Presence of serious heart, kidney, liver, gastrointestinal, infectious, endocrine disease or cancer; (iv) Presence of history of neurological disorders (e.g., traumatic brain injury, stroke, Parkinson's disease, multiple sclerosis) or have signs of cognitive decline (e.g., mild cognitive impairment, dementia); (v) treatments that would affect sleep; (vi) abnormal conventional brain MR imaging such as tumors or subdural hematomas.

Healthy controls were age-, sex-, and education-level-matched to patients, and were recruited from the community by using advertisements. Inclusion criteria for healthy controls were as follows: (i) right-hand dominance; (ii) good sleep quality and regular sleep habits; (iii) no history of chronic neurological or psychiatric disorders and medications abuse; (iv) normal conventional brain MR imaging.

In this study, there were 30 patients with primary insomnia and 30 healthy controls who fulfilled the inclusion criteria. Brain imaging data were acquired from all participants. Among these participants, two of 30 PI patients and one of 30 healthy controls were excluded because of incomplete MRI data. Two patients and one healthy control were excluded due to excessive head motion during MRI scanning. All participants completed a packet of questionnaires including demographic data, Pittsburgh sleep quality index (PSQI), Self-rating Anxiety Scale (SAS), and the Self-rating Depression Scale (SDS). Besides, patients with primary insomnia also were asked to complete Insomnia Severity Index (ISI).

### MRI data acquisition

MRI data were acquired using a Siemens 3.0 Tesla scanner (Skyra, Siemens, Erlangen, Germany) in the Department of Radiology for Beijing Hospital of Traditional Chinese Medicine Affiliated to Capital Medical University. Participants were placed in a supine position with their head snugly fixed by straps and foam pads to minimize head movement. All participates were instructed to stay awake, keep still with their eyes closed, and remain as motionless as possible. A high-resolution T1-weighted magnetization prepared rapid gradient echo (MP-RAGE) sequence was acquired and covered the entire brain [192 sagittal slices, repetition time (TR) = 2,300 ms, echo time (TE) = 2.32 ms, field of view (FOV) = 240 × 240 mm, flip angle = 8°, acquisition matrix = 256 × 256, 290 scans]. Rs-fMRI data were acquired using a singleshot gradient echo-planar imaging (EPI) sequence (40 axial slices, TR = 3,000 ms, TE = 30 ms, FOV = 220 × 220 mm, flip angle = 90°).

### Imaging data preprocessing

Image preprocessing was performed using the Resting State fMRI toolbox (DPARSF) (Chao-Gan and Yu-Feng, 2010) and SPM8 (http://www.fil.ion.ucl.ac.uk/spm/software/spm8) for MATLAB. The preprocessing of functional images is as the following main steps.

The first 10 volumes of each participant were discarded to allow the participants to adapt to the magnetic field, leaving 240 volumes for further analyses. The differences of slice acquisition times were corrected using slice timing and realigned to correct for head motions using the least-squares minimization. Participants with head motion of more than 3 mm maximum displacement in any direction, x, y, or z, or 3° of any type of angular motion were excluded. Spatial normalization was conducted referencing to a standard brain template in the Montreal Neurological Institute coordinate space, resampling to 3 × 3 × 3 mm^3^. The effects of nuisance signals including white matter signals, cerebral spinal fluid signal, and Friston 24 head motion parameters were regressed. To reduce the effect of low-frequency drift and high-frequency noise, the fMRI wave form of each voxel was temporally band-pass filtered (0.01 ≤ f ≤ 0.08 Hz), and the linear trend of the time series were removed. These images were smoothed with a 6 mm full-width half-maximum Gaussian kernel.

### VMHC analysis

VMHC was computed with the DPARSF toolbox. Homotopic resting-state FC was computed as the Pearson correlation coefficient between each voxel's residual time series and that of its symmetrical inter-hemispheric counterpart. The computed correlation coefficients were then Fisher's r-to-z transformed to get normalized z-map data. The resultant values constituted the VMHC and were used for the group analyses.

### Seed-based FC

Seed-based FC was performed using a temporal correlation approach (Fox et al., 2005). Brain regions showing significant differences in VMHC maps between the PI patients and healthy controls were defined as seed regions. Correlation analyses were conducted between the seeds and all contralateral hemispheric voxels. Correlation coefficients were then converted to z values using Fisher's r-to-z transformation to improve the normality.

### Statistical analysis

Demographic data and clinical characteristics were analyzed by using the SPSS 22 (IBM Corporation, Armonk, NY, USA). Shapiro-Wilk test was used for all study variables to determine if they follow the normal distribution. A parametric statistical test was used if the data had a normal distribution. Otherwise, the data were evaluated using a non-parametric statistical test. The chi-square test was used to compare the gender ratios. A two-sample *t*-test and Mann–Whitney U statistic were used to assess the differences in baseline age, education year, PSQI, SAS, and SDS.

Group comparisons of global VMHC were analyzed using two-sample *t*-test adjusting for age, gender, education level. The AlphaSim procedure was implemented in the DPARSF software and used for multiple comparison correction (corrected with a single voxel height of *P* < 0.01; cluster level *P* < 0.05, cluster volume > 1,296 mm^3^). The VMHC values of the brain regions that showed abnormal interhemispheric connectivity were extracted. Mean VMHC values were used for receiver operating characteristic curves (ROC) analysis. For each seed, two-sample *t-*test was used to detect the voxels showing significantly different FC with the seed between two groups adjusting for age, gender, education level (AlphaSim corrected in the mask with significant seed-based FC in both group, a single voxel height of *P* < 0.01; cluster level *P* < 0.05, cluster volume > 1647 and 2025 mm^3^, respectively). Pearson's correlation analysis was performed to ascertain the relationships between abnormal VMHC values or seed-based FC values and clinical characteristics in PI patients, including PSQI, SAS, SDS, and insomnia duration.

## Results

### Demographic and clinical characteristics

As shown in Table 1, 26 patients (mean age: 48.85 ± 12.02 years, 15 females) and 28 healthy controls (mean age: 49.07 ± 11.81 years, 16 females) were included in the final analysis. There were no significant differences in sex, age, and education level between the PI group and the healthy control group (*P* > 0.05). PI group had higher PSQI, SAS, and SDS scores than healthy controls (*P* < 0.001).

**Table 1:** Demographic and clinical characteristics of PI patients and healthy controls.

**Parameter**	**PI patients**	**Healthy controls**	***P*-value**
Sex			
Male	11	12	0.59
Female	15	16	
Age (year)	48.85 ± 12.02	49.07 ± 11.81	0.71
Education (year)	12.50 ± 4.25	13.58 ± 3.29	0.56
Insomnia duration (month)	17.62 ± 3.80	N/A	
PSQI	15.73 ± 2.09	2.58 ± 1.17	0.000
ISI	14.38 ± 2.48	N/A	
SAS	41.81 ± 8.65	30.42 ± 5.15	0.000
SDS	45.51 ± 10.17	33.81 ± 6.85	0.000

*Data are mean ± standard deviation (mean ± standard error for insomnia duration). PSQI, Pittsburgh sleep Quality index; ISI, insomnia severity index; SAS, self-rating anxiety scale; SDS, self-rating Depression scale; N/A, not applicable*.

### VMHC differences between groups

**Figure 1:**
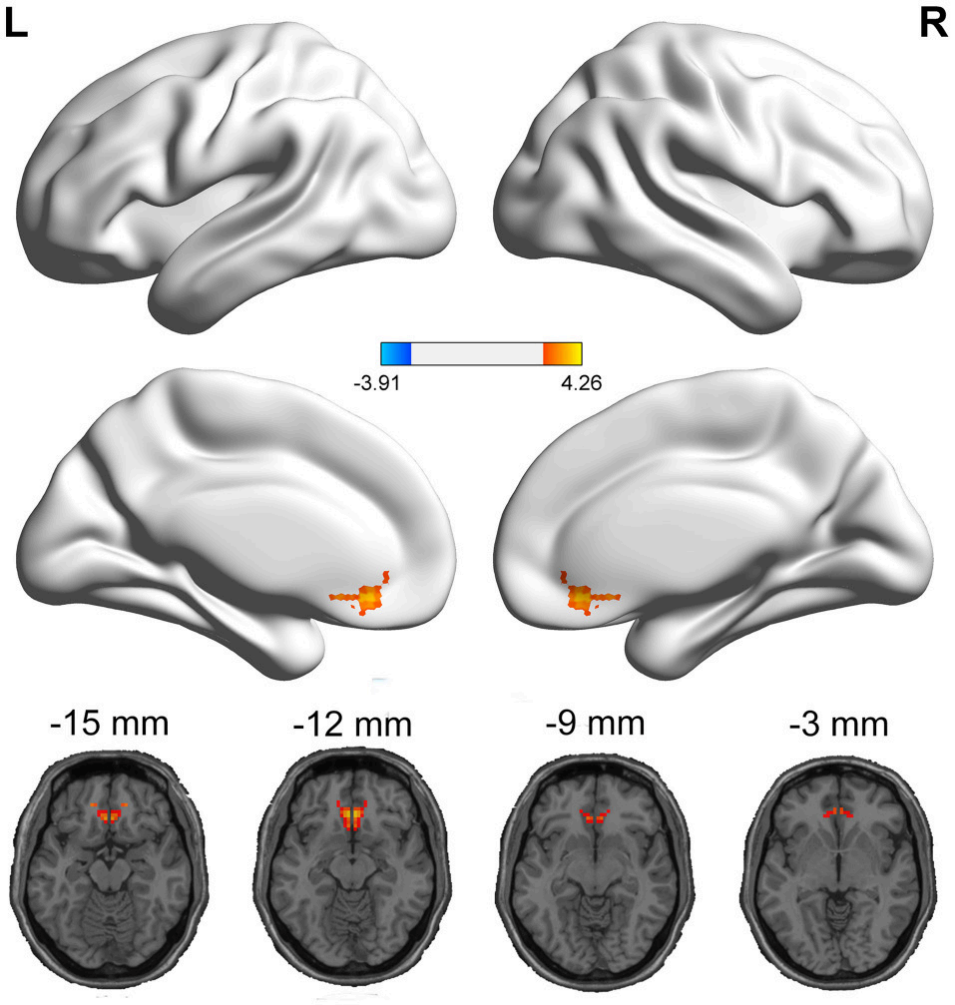
Medial views and axial medial views of significant changes in VMHC between PI group and healthy control group. The effects are significant at single voxel *p* < 0.01, AlphaSim corrected cluster level *p* < 0.05. The hot color indicates that the PI group had increased VMHC compared with the healthy control group.

The PI group had significantly increased VMHC values in the ACC, when compared with the healthy control group (Figure 1 and Table 2). No regions exhibited decreased VMHC values in the PI group, relative to the healthy control group.

**Table 2:** Brain regions showing VMHC and seed-based functional connectivity differences between two groups.

**Brain regions**	**Side**	**MNI coordinates**			**Cluster size (mm3)**	**Peak *t* value**
		**x**	**y**	**z**		
**VMHC DIFFERENCES, PI PATIENTS > HEALTHY CONTROLS**						
Anterior Cingulate Cortex	Left	3	33	−12	1,431	3.94
Anterior Cingulate Cortex	Right	−3	33	−12	1,431	3.94
**SEED-BASED RESTING-STATE FUNCTIONAL CONNECTIVITY: THE RIGHT ACC, PI PATIENTS > HEALTHY CONTROLS**						
Anterior Cingulate Cortex	Left	0	33	−9	3,942	4.31
Orbitofrontal cortex	Left	−21	48	−21	2,808	4.00
**SEED-BASED RESTING-STATE FUNCTIONAL CONNECTIVITY: THE LEFT ACC, PI PATIENTS > HEALTHY CONTROLS**						
Thalamus	Right	12	0	18	1,701	4.13
Anterior Cingulate Cortex	right	9	33	−12	5,481	4.27

*MNI, Montreal Neurological Institute*.

Since the ACC exhibited significant VMHC differences between the PI group and the healthy control group, it was used as a biomarker to separate PI patients from healthy controls. To test this possibility, mean VMHC values were extracted from the left ACC and ROC analysis was conducted. The areas (Supplementary Figure 1) under the curves (AUC) of the ACC were relatively high (AUC: 0.76, 95% CI: 0.63–0.89, p = 0.001).

### Seed-based functional connectivity between groups

**Figure 2:**
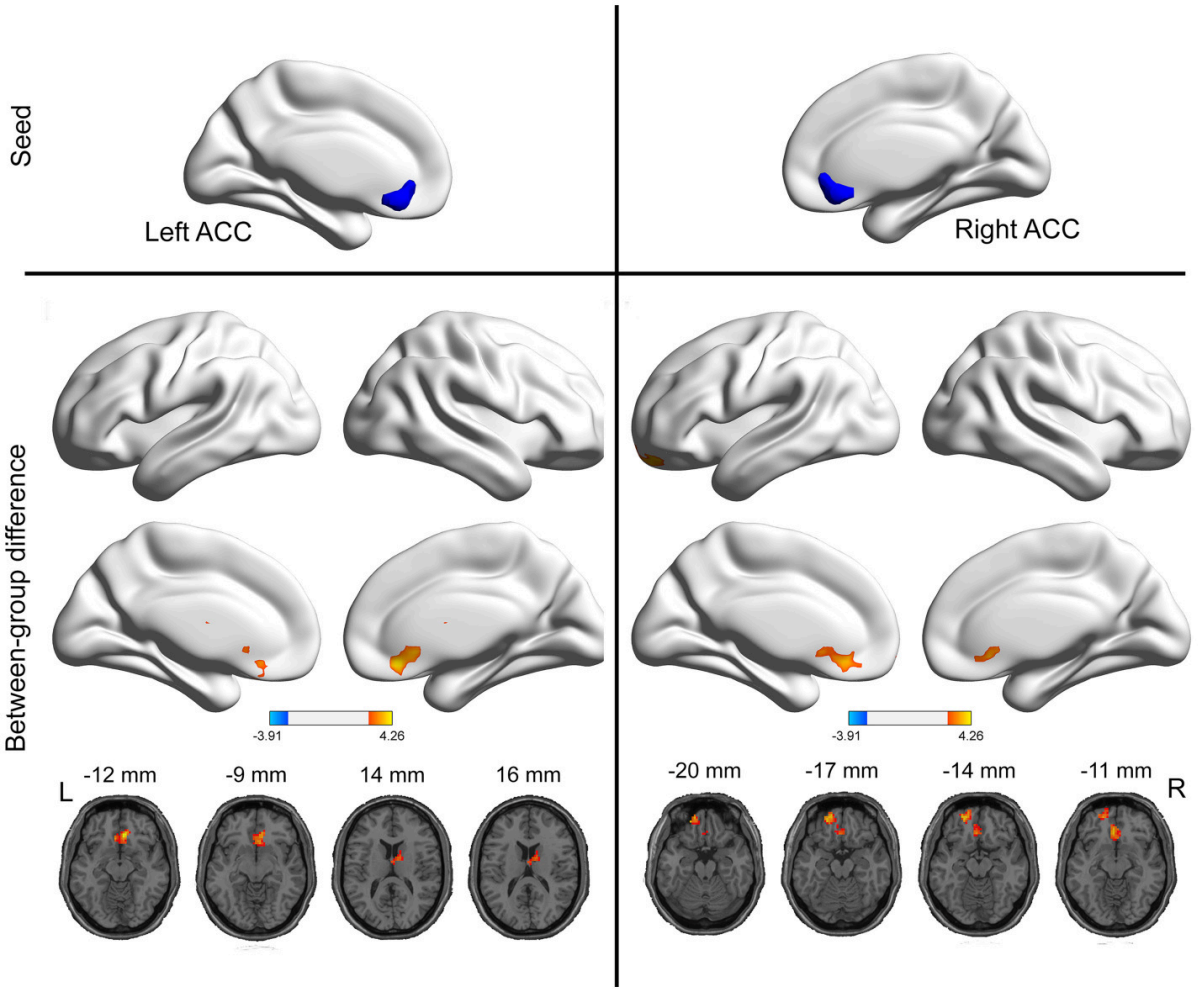
Group differences in seed-based functional connectivity. The seeds were defined as the right and left ACC. The hot color represent increased functional connectivity in the PI group compared to healthy control group. The effects are significant at single voxel *p* < 0.01, AlphaSim corrected cluster level *p* < 0.05.

As mentioned above, the ACC exhibited higher VMHC values in the PI group compared to healthy control group. Then, we examined the seed-based resting-state FC associated with two seed regions (one ACC seed per hemisphere). The seed-based FC analysis revealed increased FC between the right ACC and left orbitofrontal cortex, as well as increased FC between the left ACC and right thalamus, in PI group than healthy control group (Figure 2 and Table 2). No regions exhibited decreased FC in PI group compared with healthy control group.

### Relationships between VMHC and clinical characteristics

**Figure 3:**
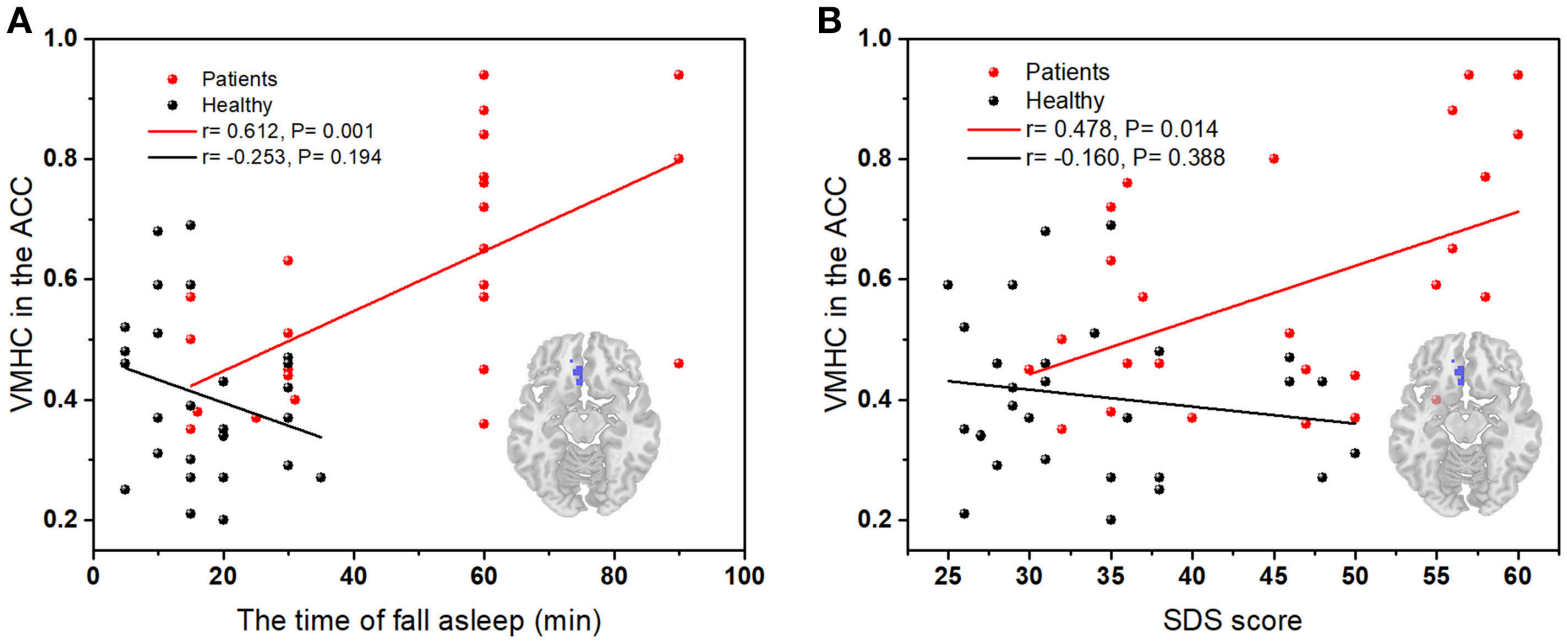
**(A)** The correlation between the time of asleep and VMHC values in the left ACC (peak coordinate: 3, 33, −12); **(B)** The correlation between SDS scores and VMHC values in left ACC (peak coordinate: 3, 33, −12); Red dots, PI group; black dots, healthy control group.

In Figure 3A, there is a significant positive correlation between the values of VMHC in left ACC and the time of fall asleep (the subitem in PSQI). The VMHC values in Figure 3B showed an obvious positive correlation with SDS scores. There was no significant correlation between the FC values and other clinical characteristics including SAS, total scores of PSQI, and insomnia duration.

## Discussion

The primary finding of this study was the significant increase in the homologous resting-state FC of the ACC in PI patients. The increased FC between the left ACC and right thalamus, and between the right ACC and left orbitofrontal cortex were also found. These results indicate that the PI patients exhibit abnormal functional coordination between left and right hemispheres. Further analysis revealed significant positive correlations between VMHC values in the left ACC and the time of fall asleep or SDS score in PI patients, suggesting that the abnormal homologous FC is also behavioral relevant.

Accumulating evidence over the past decades indicated that the ACC may be involved in the neurobiology of insomnia (O'byrne et al., 2014). In the present study, we found an abnormal interhemispheric homotopic resting-state FC in the ACC. This finding is consistent with a previous study in which a significant increased VMHC in the ACC in 27 healthy participants with insomnia symptoms was found (Li X. et al., 2017). Although current understanding of the underlying mechanisms remains limited, the construct that has been mostly used to explain the etiology of insomnia is hyperarousal (Chen et al., 2017). Emotional experience can contribute to a state of chronic hyperarousal in insomniacs (Wassing et al., 2016). The ACC, a part of the emotional circuits, has been thought an important area, in which functional differences in primary insomnia are expressed (Winkelman et al., 2013; Wang et al., 2017). In a positron emission tomography study, patients with insomnia showed reduced relative waking metabolism in the ACC than that in the controls (Nofzinger et al., 2004). This finding was corroborated by proton magnetic resonance spectroscopy studies. Morgan and Spiegelhalder also observed that PI subjects exhibited reduced GABA in this region compared with that of the controls (Morgan et al., 2012; Spiegelhalder et al., 2016). Using regional homogeneity (ReHo), Wang et al. found increased ReHo in the right ACC in PI patients than the controls (Wang et al., 2016). A morphometric analysis has revealed that ACC volumes in PI samples are significantly larger than healthy controls. Both two separated and independent studies demonstrated increases in normalized ACC volume in PI patients compared with controls, and found that bilateral ACC were the primary areas of interest investigated in PI patients (Winkelman et al., 2013). Agreed with prior studies, our study reveals the sensitivity and specificity of the VMHC values in the ACC by using ROC analysis, which means that insofar as the VMHC values in the ACC might be used as a biomarker to separate the PI patients from healthy controls.

Notably, correlation analysis showed a significant positive correlation between the VMHC values in the ACC and the time of fall asleep in the PI group. This suggests that higher interhemispheric communication in the ACC may cause the longer time of fall asleep in the PI patients. Previous studies have observed that the volume in the ACC was positively correlated with sleep onset latency, and the larger volume in this region was associated with worse sleep (Winkelman et al., 2013). Generally, patients with insomnia often have considerable anxiety about sleep, such as being unable to sleep (Riemann et al., 2010). Human and animal lesion studies have produced a wealth of data about the role of the ACC in the processing of anxiety and fear (Bush et al., 2000; Etkin et al., 2011). Thus, we speculated that the increased VMHC values in the ACC in the present study might indicate the heightened worries in PI patients. Meanwhile, insomnia sufferers have a significantly higher risk of developing depression (Baglioni et al., 2011). Interestingly, there is a significant positive correlation between the VMHC values in the ACC and SDS scores in the PI group. Depressive symptoms are more common in PI patients, and the ACC is also a key region associated with depressive symptoms (Bush et al., 2000). Sleep disorders are common with major depression, and 90% of depressed subjects have complaints related to their sleep (Bjørngaard et al., 2011; Hein et al., 2017). The hyperarousal model assumes that the experience of chronic insomnia may have a decisive impact on the development of relevant psychopathology such as depression and anxiety disorders (Riemann et al., 2010). Our research results are compatible with the notion.

Li et al. computed VMHC value in healthy participants with insomnia symptoms, but some different results can be found between this study and our study. The Li et al. study showed increased VMHC in several brain regions including ACC, bilateral thalamus/posterior insula, fusiform, middle cingulate gyrus, inferior parietal lobe, and postcentral gyrus. However, our study demonstrated VMHC increases mainly lie in the ACC. This may be related to the data methodology and the study population. First, the significance levels were set at *P* < 0.05 (combined height threshold *P* < 0.05 and a minimum cluster size was 85) in the previous study. A multiple testing correction approach was used in our study in order to better control a high inflated false positive rate, and the significance levels were set at *P* < 0.01. If we adopt the method of Li et al., patients with PI showed differences in VMHC in several brain regions, including the parahippocampal cortex, fusiform cortex, temporal inferior cortex, occipital lobe, and anterior cingulate cortex, when compared with healthy controls. Those brain regions are close to the previous study. Second, the crucial cause for the difference lies in the selected patients in the two studies. The prior study recruited the healthy participants with insomnia symptoms. The sleep disturbance scores were determined by the sum of 3 HAMD-17 sleep questions, including items 4 (insomnia-early), 5 (insomnia-middle), and 6 (insomnia-late). Though the HAMA also contains insomnia-related items (i.e., items 4), it cannot be considered as the main objective measure of insomnia. It is obvious that no professional sleep-related assessment scales were used to measure insomnia symptoms. On the contrary, the PI patients enrolled in our study were all diagnosed as primary insomnia by the doctors of Department of Psychosomatic Medicine. Professional sleep-related assessment scales, such as PSQI and ISI, were measured in our study.

In this study, we demonstrated an increase FC in the right ACC with left orbitofrontal cortex in PI patients. Insomnia is also hypothesized as a disorder of sleep-wake regulation (Buysse et al., 2011). Prefrontal dopamine levels are involved in the regulation of sleep and wakefulness in normal subjects (Dauvilliers et al., 2015). Activity in the ACC, which is functionally related to the prefrontal cortex, plays an associative or executive role in decreasing the cerebral blood flow during slow wave (Braun et al., 1997). To date, the MRI sleep literature has most frequently evaluated the associations between the orbitofrontal cortex changes and sleep quality (Sexton et al., 2014). A voxel-based morphometric study demonstrates that elderly insomniacs have a significantly reduced gray matter (GM) density in part of the left orbitofrontal cortex (Altena et al., 2010). More importantly, reduced GM density in left orbitofrontal cortex is likely to represent pre-existing vulnerability to sleep complaints, rather than a consequence of insomnia (Stoffers et al., 2012). Consistently, fMRI studies have shown decreased amplitude of low-frequency fluctuations (ALFF) in the bilateral orbitofrontal cortex after sleep deprivation (Gao et al., 2015). In addition, lower ALFF value in the left orbitofrontal cortex was also observed after experiencing a period of insomnia, and the duration of PI negative correlated with ALFF value in the left orbitofrontal cortex (Li C. et al., 2016).

Our current study found that the left ACC revealed significant enhancement of FC with the right thalamus in PI group. Generally, the thalamus crucially relates to the generation of wakefulness (Scammell et al., 2017). At the level of brain structures and molecules, wakefulness is governed by neurons in the upper brainstem projecting to higher brain areas, and monoaminergic neurons and cholinergic neurons provide input to the thalamus, which plays an important role in the maintenance of wakefulness (Saper et al., 2010). An animal study showed widespread proteomic alterations in the thalamic synapses due to sleep deprivation (Simor et al., 2017). Similarly, patients, with lesions just limited to the thalamus, are usually in an awake state (Posner et al., 2007). Patients developed sleep disorders after acute thalamic stroke or acute insomnia following surgery of the ventralis intermedius nucleus of the thalamus for tremor (Quigg et al., 2007; Li Q. et al., 2017). Recent neuroimaging studies have also identified the cerebral mechanisms of insomnia pathogenesis. For example, the thalamus exhibits increased interhemispheric synchrony in healthy adults with insomnia symptoms (Li X. et al., 2017). To avoiding external perturbations, the information is selected by the thalamus to project to the asleep cortex (Del Felice et al., 2012). By using the ALFF method, reduced spontaneous neuronal activity was found in the thalamus in healthy adults with insomnia symptoms (Liu et al., 2016). The thalamus forms an important relay between sensory input and the cerebral cortex, that is, specific sensory information is input to the thalamus and further sent to specialized cortical areas via thalamocortical projections (Reislev et al., 2017). Significant changes in thalamic connectivity with the cortical cortex were also observed after sleep deprivation (Lei et al., 2015; Yeo et al., 2015; Xu et al., 2017). Consistent with previous research, our findings demonstrated the abnormal FC in the thalamus and provided the evidence to support the hypothesis that insomnia associated with hyperarousal (Liu et al., 2016).

We are aware of several methodological limitations in our current study. First, the current study did not include a large sample size, which may attenuate the statistical power in detecting the between-group differences in some of our measures. Future studies with larger sample sizes are needed to confirm the reliability of the observed effects. Second, our study is cross-sectional, not longitudinal. Longitudinal imaging studies with treatment are essential to confirm the causal relationship of the VMHC and insomnia. Third, although neuroimaging studies demonstrate increased levels of arousal in primary insomnia during both night and daytime, the findings in the present study should be regarded as exploratory in nature because no fMRI date was obtained in the evening when the sleep occurred. Further studies that collect fMRI dynamics of wakefulness and deep sleep will contribute to our better understanding the etiology and pathogenesis underlying this disorder. A previous study showed some regions exhibited increased homotopic FC with age (Zuo et al., 2010b). The age of the patients recruited in our study ranged from 25 to 60 years. Although we analyzed the data adjusting for age, further studies with a small age span are need.

In summary, our study illustrated PI patients display abnormal FC compared with healthy controls, not only in VMHC in the ACC, but also seed-based FC between the ACC and other brain regions. The current study provides completed evidence for interhemispheric disconnection associated with primary insomnia, and support that enhanced FC in the ACC is involved in pathology mechanism of primary insomnia.

## Author contributions

Q-QL conceived and designed the experiments. J-WH, PZ, Q-NF, and JZ performed the experiments. XW and SZ analyzed the data. C-QY and C-ZL wrote the paper. All authors approved the final manuscript.

### Conflict of interest statement

The authors declare that the research was conducted in the absence of any commercial or financial relationships that could be construed as a potential conflict of interest. The reviewer BS and handling Editor declared their shared affiliation.

## Supplementary material

Supplementary Figure 1 Receiver operating characteristic curves by using the VMHC values in the ACC and the mean VMHC value in left ACC separating patients with primary insomnia from healthy controls.
