# Supplementary figures and images for: Abnormal Functional Connectivity of Anterior Cingulate Cortex in Patients With Primary Insomnia: A Resting-State Functional Magnetic Resonance Imaging Study
Source: Front Aging Neurosci. 2018 Jun 5;10:167. doi: 10.3389/fnagi.2018.00167 (PMC5996039; doi:10.3389/fnagi.2018.00167)

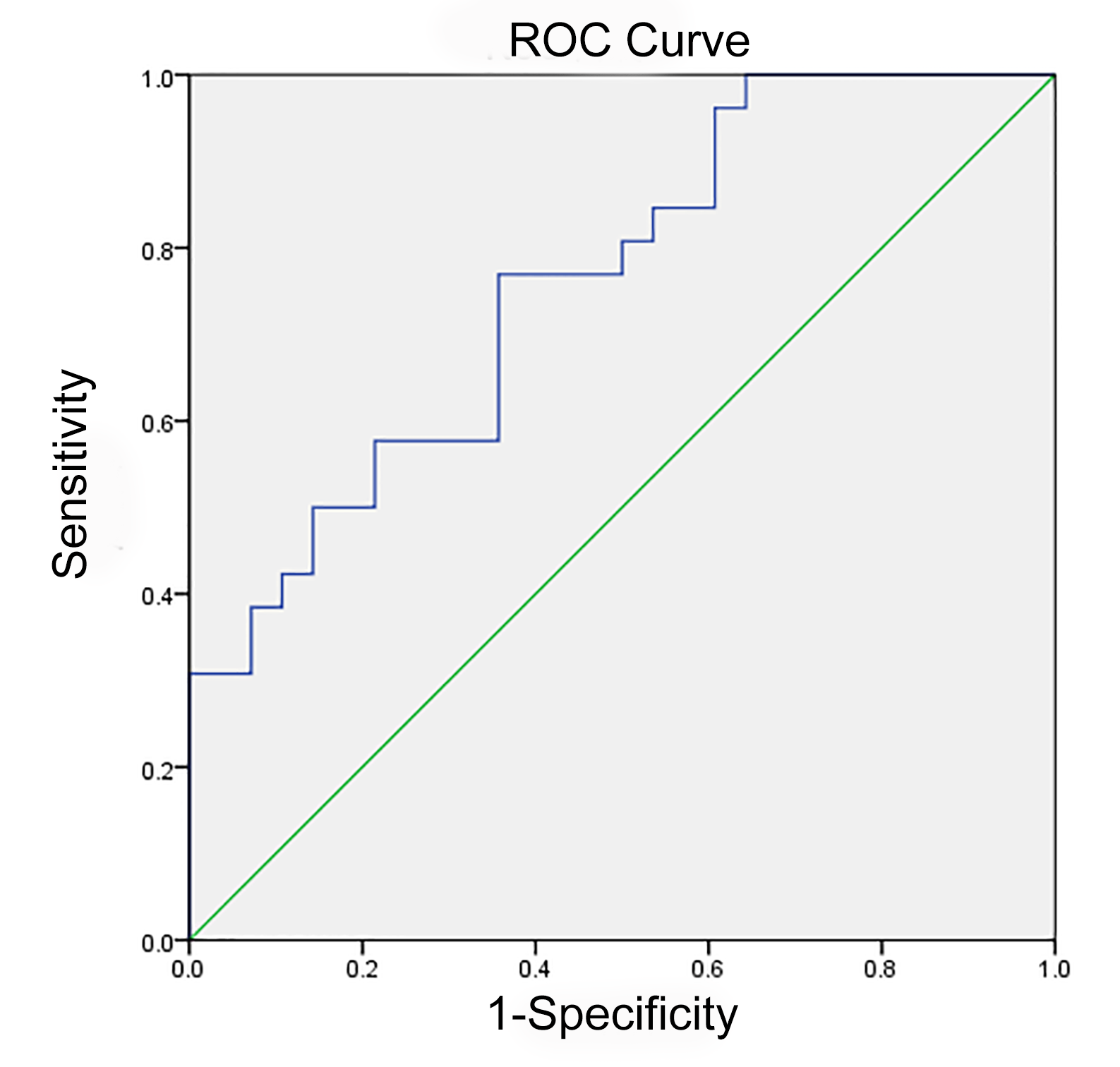

Supplement: Supplementary Figure 1 — Receiver operating characteristic curves by using the VMHC values in the ACC and the mean VMHC value in left ACC separating patients with primary insomnia from healthy controls. [file Image_1.TIF]
